# Meta-analysis of the prognostic value of circulating tumor cells in gastrointestinal cancer

**DOI:** 10.1097/MD.0000000000031099

**Published:** 2022-10-21

**Authors:** Yuming Yao, Xiang Zhu, Weixin Liu, Jiayi Jiang, Han Jiang

**Affiliations:** a Jiangxi Provincial Key Laboratory of Preventive Medicine, School of Public Health, Nanchang University, Nanchang, China; b Mathematics Major, New York University, New York, NY, USA; c Department of Thoracic Surgery, The Second Affiliated Hospital of Nanchang University, Nanchang, China.

**Keywords:** circulating tumor cells, colorectal cancer, esophageal cancer, gastric cancer, gastrointestinal tumors, meta-analysis, prognosis

## Abstract

Detecting circulating tumor cells (CTCs) has become a new strategy for predicting the prognosis of cancer patients. However, limited systematic research evidence is available for the detection of CTCs in various gastrointestinal tumors such as esophageal cancer (EC), colorectal cancer (CRC) and gastric cancer (GC). This topic was addressed to assess the prognostic significance of CTCs in gastrointestinal tumors.

**Methods:**

We conducted a literature search according to the Preferred Reporting Items for Systematic Reviews and Meta-Analyses (PRISMA) checklist (from November 20, 2021). We performed a meta-analysis using the random effects model and Review Manager 5.3 software (The Cochrane Collaboration, Copenhagen, Denmark) according to the inclusion and exclusion criteria, data extraction and evaluation methods.

**Results:**

Twenty-four articles met the inclusion criteria for this study, and they included 3803 EC, CRC and GC patients, including 1189 CTC-positive and 2462 CTC-negative cases. The meta-analysis showed that the presence of CTCs was associated with worse OS (HR = 2.05, 95% CI = 1.75–2.40, *P* = .060) and PFS (HR = 2.27, 95% CI = 1.79–2.89, *P* < .001). Further meta-regression and subgroup analyses showed that CTC-positive patients also showed worse OS and PFS in different subgroups.

**Conclusion:**

Our meta-analysis suggests that detecting CTCs in peripheral blood may be an important tool for improving the prognosis of patients with gastrointestinal tumors. Moreover, CTCs detection results could be used to develop personalized treatment plans in the future.

## 1. Introduction

To date, gastrointestinal tumors are still the main malignant tumors, among which gastric cancer (GC), colorectal cancer (CRC) and esophageal cancer (EC) are the third, fifth and seventh most common malignant tumors, respectively.^[1]^ In 2020, new cases of EC, GC and CRC worldwide were estimated at 604,110, 1,089,203 and 1,931,590, respectively. The incidence rates of EC and GC in China are among the highest in the world.^[2]^ To date, the pathological stage, histological type, and lymphatic and vascular invasion have been widely used as prognostic factors for patients with gastrointestinal cancer; however, these parameters have certain limits.^[3]^

Circulating tumor cells (CTCs) were first proposed by Professor Ashworth in 1896 and defined as tumor cells that detach from the primary tumor and then enter the blood circulation.^[4]^ Today, CTCs have been used in many aspects of cancer management, such as recording tumor recurrence and treatment effects, determining drug selection strategies, and predicting cancer patient survival.^[5]^ Many studies have applied CTCs in peripheral blood to prognosis evaluations of human cancers, such as GC,^[6]^ head and neck squamous cell carcinoma,^[7]^ prostate cancer,^[8]^ EC^[9]^ and breast cancer.^[10]^ Allard^[11]^ reported the detection of CTCs in patients with prostate cancer, breast cancer, CRC, and other gastrointestinal cancers. In addition, the CTCs count is also closely related to overall survival (OS), progression-free survival (PFS) and disease-free survival (DFS). The technologies commonly used to detect CTCs mainly include CellSearch, reverse transcription polymerase chain reaction (RT-PCR) and flow cytometry, among which CellSearch is the first and only FDA-approved method of recording colorectal, stomach, breast, and prostate cancer.^[12]^

Although studies^[3,13,14]^ have suggested that the number of CTCs at baseline has important prognostic value in esophageal, colorectal, and GCs, systematic evidence for multiple gastrointestinal tumors is lacking. To better understand the prognostic value of CTCs in patients with gastrointestinal tumors (esophageal, colorectal, and gastric), we performed a meta-analysis of published literature on this topic. In particular, we assessed the prognostic value of CTCs status (presence and absence) on the OS, PFS and DFS of patients with gastrointestinal tumors. This meta-analysis shows the role of CTCs numbers t baseline as a reliable predictor of prognosis in EC, CRC, and GC. This prognostic role of CTCs for gastrointestinal tumors will help doctors counsel patients, define and predict the cancer risk layer, and estimate the response to chemotherapy.^[15]^

## 2. Materials and Methods

### 2.1. Literature search

Two authors independently searched the PubMed, Web of Science, Cochrane Library, CNKI, Wanfang and SinoMed databases. The search deadline was November 20, 2021. The following search terms were used: circulating tumor cells, circulating cancer cells, CTCs, esophageal carcinoma, esophageal cancer, esophageal squamous cell carcinoma, ESCC, gastric cancer, gastric carcinoma, GC, colorectal cancer, colorectal carcinoma, CRC, prognostic, prognosis. The retrieval strategy is shown in Table 1. In addition, references to potentially relevant studies were reviewed to exclude duplicate studies.

**Table 1:** Search strategy.

Serial number	Search strategy
1	circulating tumor cells OR circulating cancer cells OR CTCs
2	esophageal carcinoma OR esophageal cancer OR esophageal cancer OR esophageal squamous cell carcinoma OR ESCC OR EC
3	gastric cancer OR gastric carcinoma OR GC
4	colorectal cancer OR colorectal carcinoma OR CRC
5	#2 OR #3 OR #4
6	prognostic OR prognosis
7	#1 AND #5 AND #6

### 2.2. Inclusion and exclusion criteria

Articles meeting the following criteria were included in the meta-analysis: population: EC patients, CRC patients and GC patients; intervention: CTC-positive; comparison: CTC-negative; outcome: PFS or/and OS or/and DFS as the primary outcome; design: randomized controlled trials (RCTs) or observation sex studies; samples: peripheral blood (PB) and baseline (pretreatment) samples; and effect size measure: hazard ratio (HR) and its 95% confidence interval or effect size calculator.

The exclusion criteria were as follows: review articles, letters, comments, and case reports; and studies that were unable to retrieve or calculate data of interest. To avoid duplication of studies, all included studies were carefully examined, including their authors, organization, accrual time, and patient population.

OS was defined as the time from the date of blood collection to the date of death from any cause or the date of last follow-up, and PFS was defined as the time from the date of blood collection to the date of disease progression or death from any cause.^[16]^ DFS was defined as the time interval between the date of diagnosis and the date of first recurrence or last follow-up or death, whichever came first.^[17]^

### 2.3. Data extraction and quality assessment

Two reviewers independently checked the quality of the included studies and retrieved information from all eligible studies. The following information was collected: author(s), year of publication, study population characteristics (number, sex, and age), CTCs detection rate, treatment, follow-up period, and outcome measures and their HRs. If the HRs and 95% CIs were not directly provided in the original article, then time-to-event summary data could be included in the meta-analyses.^[18]^ In addition, multivariate analysis results were extracted when possible.^[19]^ The quality of the included literature was assessed by two authors using the Newcastle–Ottawa Scale (NOS),^[20]^ with a score of 0 to 5 defined as low quality and a score of 6 to 9 defined as high quality. Disagreements between the two reviewers were resolved by discussion and consensus. If an agreement could not be reached, then an additional arbitrator was invited to participate in the discussion.

### 2.4. Statistical analysis

Review Manager 5.3 software (The Cochrane Collaboration, Copenhagen, Denmark) was used for the meta-analysis, and the heterogeneity of the literature was judged by the Cochran Q test (*P* value) and *I*^2^ statistic. For values of *I*^2^ > 50% or *P* < .1, which indicated heterogeneity in the literature, a random-effects model was selected for pooling and interval estimation of the HR^[21]^; otherwise, a fixed-effects model was used. The HR, OS, PFS, DFS, and other outcome measures were extracted from the included studies to statistically evaluate the prognostic impact of CTCs on EC, CRC, and GC. HR > 1 reflects further disease progression or additional deaths in the CTC-positive patients.^[22]^ Subgroup and meta-regression analyses were performed to explore the sources of heterogeneity and differences between groups; publication bias was assessed using funnel plots; and sensitivity analyses were performed by removing a single study from the overall pooled analysis each time and then performing the meta-analysis again to compare the magnitude of the observed change with the previous results.

## 3. Results

### 3.1. Study selection

**Figure 1.**
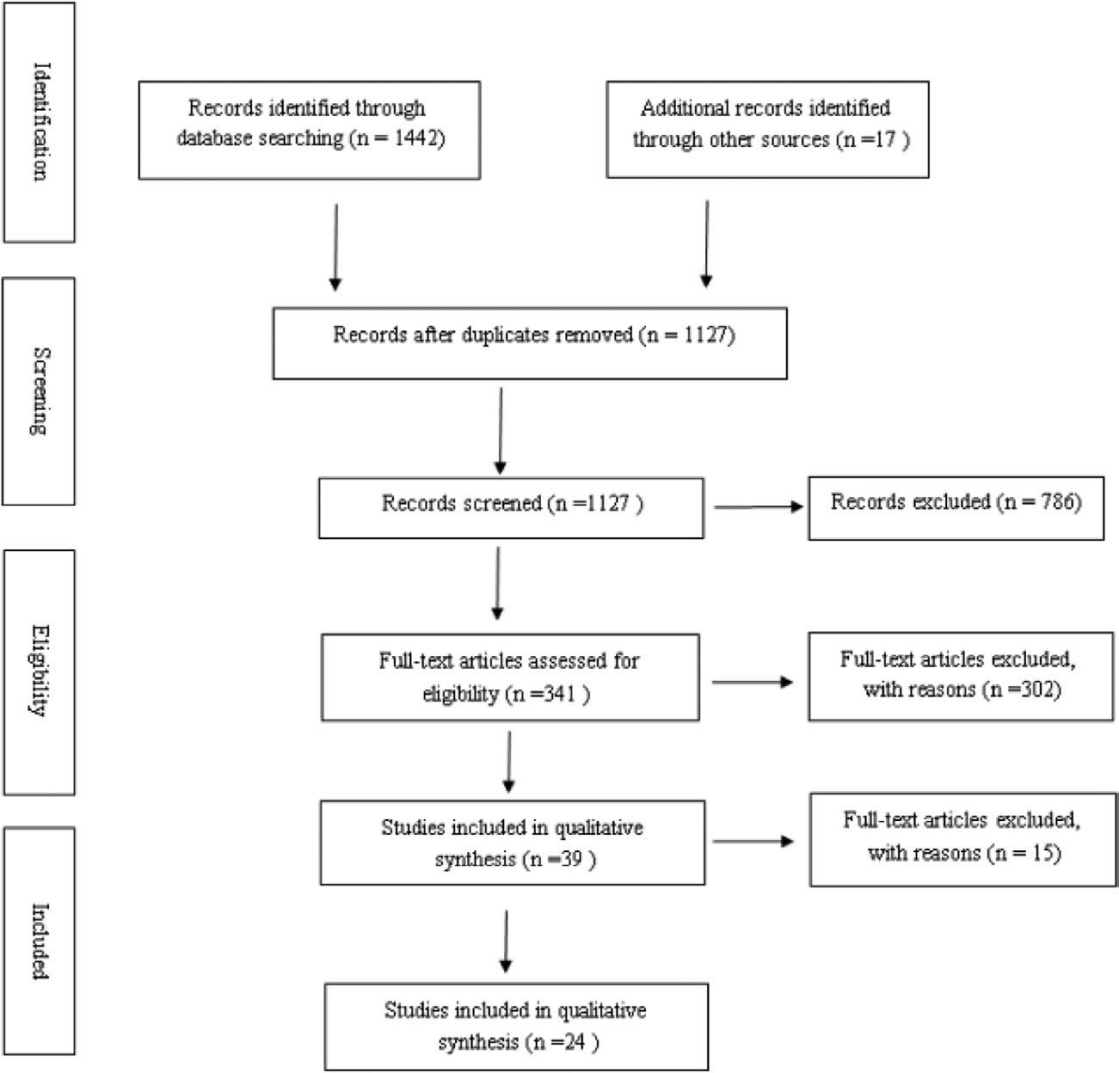
Flow charts of studies included in meta-analysis.

The PRISMA flow chart of this meta-analysis is shown in Figure 1. A total of 1459 studies were initially retrieved, and 332 studies were excluded due to duplication. After reviewing the titles and abstracts, 786 irrelevant studies were excluded. A total of 302 studies were excluded due to a lack of results of interest. After a full-text analysis of the remaining 39 relevant studies, 15 studies were excluded due to incomplete outcome data. Finally, a total of 24 relevant studies were selected for meta-analysis.

### 3.2. Study characteristics

The main characteristics of the included studies are shown in Table 2. These studies were from 11 countries (China, Japan, Korea, the USA, the UK, the Netherlands, Germany, Brazil, Spain, Norway and Italy) and included a total of 3803 patients with esophageal, colorectal and GCs, and they were published from 2009 to 2021. Five of these studies focused on EC,^[13,23–26]^ eleven focused on CRC,^[14,27–36]^ and eight focused on GC.^[16,37–43]^ The sample sizes ranged from 18 to 467 (n = 1189 in the CTC-positive group; n = 2462 in the CTC-negative group). All studies assessed baseline CTCs, 17 studies assessed the HR for PFS, 17 studies assessed the HR for OS, 2 studies assessed the HR for DFS, and 1 study assessed the dCTC (difference between the number of CTCs before treatment and the number of CTCs after treatment) HR. Methods used for CTCs detection included RT–PCR (reverse transcription polymerase chain reaction), CellSearch CTCs assay and others (ISET, CMx platform, immune-magnetic and FACS-ICC). According to the Newcastle–Ottawa scale,^[20]^ 91.7% (22/24) of these studies were of high quality (quality score ≥ 6).

**Table 2:** Baseline characteristics of the included studies.

No.	Ref.	Year	Country	Cancer type	Number (M/F)	Rate % (n/N) Baseline	Detection method	Cutoff criteria	Outcome	Quality scores
1	Yin et al^[26]^	2012	China	ESCC	72 (54/18)	69.4 (50/72)	RT-PCR	Expression of any one CEA, CK19, surviving	PFS	8
2	Matsushita et al^[13]^	2015	Japan	ESCC	90 (78/12)	27.8 (25/90)	CellSearch	≥1/7.5 mL	OS	7
3	Cao et al^[25]^	2009	China	ESCC	108 (85/23)	47.2 (51/108)	RT-PCR	Expression of surviving	OS, PFS	7
4	Tanaka et al^[24]^	2010	Japan	ESCC	244(212/32)	13.9(34/244)	RT-PCR	Expression of any one CEA, SCCA	OS, DFS	6
5	Li et al^[23]^	2020	China	ESCC	38 (28/10)	81.6 (31/38)	CellSearch	>3/3.2 mL	dCTC	7
6	Cohen et al^[29]^	2009	NL	CRC	430 (NR)	26.2 (108/413)	CellSearch	≥3/7.5 mL	OS, PFS	6
7	Tol et al^[32]^	2010	Japan	CRC	467 (284/183)	29.6 (129/451)	CellSearch	≥3/7.5 mL	OS, PFS	6
8	Aggarwal et al^[30]^	2013	USA, UL and UK	CRC	430 (238/192)	47.2 (203/430)	CellSearch	≥3/7.5 mL	OS	8
9	Bork et al^[35]^	2013	Germany	CRC	287 (186/101)	10.5 (30/287)	CellSearch	≥1/7.5 mL	OS, PFS	7
10	Gazzaniga et al^[44]^	2013	Italy	CRC	119 (68/51)	35.3 (42/119)	CellSearch	≥1/7.5 mL	PFS	7
11	Katoh et al^[27]^	2015	Japan	CRC	150 (91/59)	40.0 (60/150)	RT-PCR	Expression of CD44v9	PFS	4
12	Kuboki et al^[28]^	2013	Japan	CRC	63 (34/29)	30.2 (19/63)	CellSearch	≥3/7.5 mL	OS	7
13	Sastre et al^[34]^	2013	Spain	CRC	158 (102/56)	48.1 (76/158)	CellSearch	≥3/7.5 mL	OS, PFS	6
14	Seeberg et al^[31]^	2015	Norway	CRC	194 (105/89)	13.4 (26/194)	CellSearch	≥2/7.5 mL	OS, PFS	7
15	Silva et al^[36]^	2021	Brazil	CRC	75 (42/33)	NR	ISET	>1.5/8 mL	OS, PFS	6
16	Tsai et al^[33]^	2016	China	CRC	95 (51/44)	43.9 (36/84)	CMx platform	≥5/2 mL	DFS	7
17	Lee et al^[16]^	2015	Korea	GC	95 (63/32)	28.4 (27/95)	CellSearch	≥5/7.5 mL	OS, PFS	6
18	Sclafani et al^[38]^	2014	UK	GC	18 (16/2)	44.4 (8/18)	CellSearch	≥2/7.5 mL	PFS	4
19	Kubisch et al^[42]^	2015	America	GC	62 (39/23)	69.4 (43/62)	Immune-magnetic	≥1/10 mL	OS, PFS	7
20	Li et al^[41]^	2016	China	GC	136 (89/47)	41.9 (57/136)	CellSearch	≥3/7.5 mL	PFS	7
21	Meulendijk et al^[40]^	2016	NL	GC	60 (43/17)	66.7 (16/24)	FACS-ICC	≥2/8 mL	OS, PFS	6
22	Okabe et al^[39]^	2015	Japan	GC	136 (87/49)	18.4 (25/136)	CellSearch	≥1/7.5 mL	OS, PFS	6
23	Uenosono et al^[43]^	2013	Japan	GC	251 (170/81)	31.1 (78/251)	CellSearch	≥1/7.5 mL	OS	7
24	Huang et al^[37]^	2019	China	GC	28 (16/12)	53.6 (15/28)	CellSearch	≥4/3.2 mL	OS, PFS	6

CRC = colorectal cancer, CTCs = circulating tumor cells, DFS = disease-free survival, dCTC = difference between the number of CTCs before treatment and the number of CTCs after treatment, ESCC = esophageal squamous cell carcinoma, FACS = fluorescence-activated cell sorter, GC = gastric cancer, ICC = immunocytochemis, ISET = isolation by size of tumor cell technique, OS = overall survival, PFS = progression-free survival.

### 3.3. The relationship between CTCs and OS and PFS

OS was the outcome measure of 17 studies, which included 180 EC patients, 2104 CRC patients and 632 GC patients. The OS and PFS of EC, CRC, and GC were pooled separately, and the results showed that the OS of patients with positive CTCs at baseline was significantly lower than that of patients with negative CTCs (HR = 2.05, fixed effect 95% CI: 1.75–2.40), and weak heterogeneity was observed (*I*^2^ = 37.0%, *P* = .060). PFS was the outcome measure in 17 studies, which included a total of 2857 patients, including 442 patients with EC, 1880 patients with CRC, and 535 patients with GC. The pooled results also showed that the PFS of CTC-positive patients was significantly lower than that of CTC-negative patients (HR = 2.41, 95% CI: 1.84–3.16) using a random-effects model, and significant heterogeneity was observed (*I^2^* = 72.0%, *P* < .001).

### 3.4. Meta-regression and subgroup analysis

Due to the significant heterogeneity among studies, we performed a meta-regression analysis to investigate the potential sources (Table 3), and the analysis considered the region, detection method, CTCs positivity cutoff from baseline sample data value, CTCs positive detection rate, follow-up time, treatment modality, median patient age, tumor type, and literature score covariates. Univariate analysis showed that CTCs positive detection rate (*P* = .002) and tumor type (*P* = .007) were significant factors affecting disease OS, CTCs positive cutoff (*P* = .011) was a significant factor affecting disease PFS, while other covariates were not significantly associated with heterogeneity in the OS or PFS studies. The results of the multivariate analysis indicated that multiple variables were not significant in explaining the heterogeneity of the OS or PFS studies.

**Table 3:** Meta-regression analysis for exploring potential sources of heterogeneity.

Variables	Univariate analysis						Multivariate analysis					
	OS			PFS			OS			PFS		
	Coef	SE	*P*	Coef	SE	*P*	Coef	SE	*P*	Coef	SE	*P*
Region	0.289	0.314	.509	0.183	0.284	.519	0.434	0.623	.487	-0.150	1.126	.894
Median age	0.473	0.285	.097	0.183	0.273	.502	-0.099	0.487	.839	0.066	1.201	.956
Detection method	0.499	0.280	.075	0.038	0.361	.916	-0.138	0.788	.861	-0.983	1.460	.501
CTC positive	-0.276	0.349	.429	-0.742	0.311	.017	-1.363	0.856	.111	-1.127	1.086	.299
Treatment	-0.613	0.353	.082	-0.107	0.386	.782	0.520	0.807	.520	-0.435	1.452	.765
Detection rate	-0.668	0.211	.002	-0.039	0.308	.900	0.338	0.828	.683	0.145	0.986	.883
Follow-up time	-0.333	0.248	.179	0.208	0.403	.606	-0.768	0.587	.191	0.513	1.471	.727
Cancer type	-0.591	0.219	.007	0.381	0.297	.199	-0.228	0.395	.563	-0.405	1.233	.743
Score	--	--	--	0.379	0.709	.593	--	--	--	1.095	1.727	.526

OS = overall survival, PFS = progression-free survival, SE = standard error.

In addition, we performed a subgroup analysis to further assess the prognostic value in different subgroups (Table 4). The results showed significant prognostic effects for OS and PFS and demonstrated that baseline CTC-positive patients had a higher risk of death or progression than CTC-negative patients in all subgroups. We first evaluated the impact of CTCs positivity status on OS and PFS in different regions (Asian and non-Asian), and the results showed that the detection of CTCs had a significant prognostic impact on patients with gastrointestinal tumors in non-Asian regions (OS: HR = 2.12, 95% CI = 1.78–2.53, *P* = .530, *I*² = 0%; PFS: HR = 2.55, 95% CI = 1.73–3.74, *P* < .001, *I*² = 74.0%) but not in Asian regions. We then assessed the impact of CTCs positivity status on OS and PFS based on the detection method and found that CTCs detected using methods other than CellSearch could predict more severe disease survival and progression (OS: HR = 2.67, 95% CI = 1.63–3.11, *P* = .003, *I*² = 72.0%; PFS: HR = 3.31, 95% CI = 1.81–6.06, *P* < .001, *I*² = 78.0%). In addition, we determined the effect of CTCs status on OS and PFS in terms of treatment and found that for patients undergoing surgery, the detection of CTCs at baseline was associated with an increased risk of poor prognosis (OS: HR = 2.25, 95% CI = 1.51–4.75, *P* = .200, *I*² = 32.0%; PFS: HR = 3.69, 95% CI = 1.99–6.87, *P* = .020, *I*² = 70.0%), although the finding did not reach statistical significance. In other subgroup analyses that included different conditions, such as different CTCs positivity cutoffs, different CTCs positivity detection rates, different median follow-up times, different median patient ages, different tumor types (EC, CRC or GC) and different literature quality scores, CTCs detection presented prognostic value for disease survival and progression in patients with gastrointestinal tumors.

**Table 4:** Results of OS and PFS subgroup analysis.

Variables	OS				PFS			
	HR [95%CI]	n	*I*²[%}	*P*	HR [95%CI]	n	*I*² [%}	*P*
Region								
Asia	2.01 [1.60–2.51]	9	58.0	.020	2.12 [1.54–2.90]	8	69.0	.002
Non-Asia	2.12 [1.78–2.53]	8	0.0	.530	2.55 [1.74–3.74]	9	74.0	<.001
Detection method								
CellSearch	2.05 [1.81–2.33]	11	0.0	.810	2.02 [1.58–2.60]	10	68.0	<.001
Non-CellSearch	2.67 [1.51–4.75]	6	72.0	.003	3.31 [1.81–6.06]	7	78.0	<.001
CTC-Positive n ≥ 3								
Yes	1.85 [1.53–2.24]	7	49.0	.070	1.59 [1.34–1.90]	6	44.0	.120
No	2.42 [1.90–3.08]	10	10.0	.350	3.21 [2.28–4.53]	11	52.0	.020
Detection rate (%)								
≥35	2.17 [1.42–3.33]	6	71.0	.004	2.54 [1.67–3.86]	9	72.0	<.001
<35	2.16 [1.88–2.48]	11	0.0	.960	2.38 [1.70–3.35]	8	77.0	<.001
Median follow-up								
≥24 mo	1.72 [1.34–2.21]	5	0.0	.670	3.02[1.42–6.46]	4	80.0	.002
<24 mo	2.21 [1.80–2.70]	12	49.0	.030	2.12 [1.67–2.68]	13	66.0	<.001
Therapy method								
Surgery	2.25 [1.63–3.11]	6	32.0	.200	3.69 [1.99–6.87]	4	70.0	.020
Non-surgery	1.99 [1.66–2.39]	11	42.0	.070	1.94 [1.57–2.40]	13	59.0	.004
Median age								
≥65yr	2.21 [1.87–2.60]	8	0.0	.770	2.25 [1.65–3.07]	8	58.0	.020
<65yr	1.94 [1.48–2.55]	9	56.0	.020	2.42 [1.66–3.54]	9	80.0	<.001
Cancer type								
ESCC	3.18 [2.10–4.82]	3	1.0	.360	4.53 [2.48–8.28]	2	0.0	.590
CRC	2.10 [1.82–2.42]	8	0.0	.850	2.27 [1.66–3.11]	8	76.0	<.001
GC	1.78 [1.28–2.48]	6	49.0	.080	2.06 [1.37–3.09]	7	67.0	.006
Quality score								
≥6	2.05 [1.75–2.40]	17	37.0	.060	2.31 [1.82–2.95]	15	73.0	<.001
<6	---	0	---	---	2.21 [0.54–8.97]	2	74.0	.050
Overall	2.05 [1.75–2.40]	17	37.0	.060	2.27 [1.79–2.89]	17	72%	<.001

CRC = colorectal cancer, ESCC = esophageal squamous cell carcinoma, GC = gastric cancer, HR = hazard ratio, OS = overall survival, PFS = progression-free survival, SE = standard error.

### 3.5. Sensitivity analysis and publication bias

**Figure 2.**
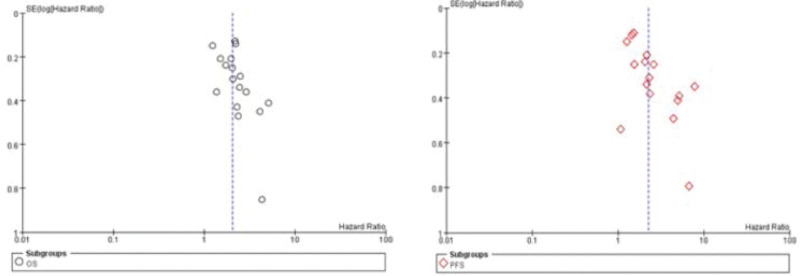
The funnel plots of OS and PFS. OS = overall survival, PFS = progression-free survival.

A sensitivity analysis was conducted by deleting one single study from the overall pooled analysis each time, and the results showed that the direction and magnitude of the OS and PFS estimates of pooled results were not significantly affected, indicating that no single study dominated our results. These data indicated that our results were stable and reliable. We used funnel plots to detect publication bias, as shown in Figure 2. The shapes of the funnel plots were symmetrically distributed in all comparisons. Therefore, significant publication bias was not found in the meta-analysis of OS and PFS.

## 4. Discussion

Gastrointestinal tumors are an important contributor to today’s global cancer burden, and they present high morbidity and mortality worldwide.^[1]^ Although great progress has been made in the treatment of EC, CRC, and GC, the five-year survival rate of patients is still low^[44,45]^ and tumor metastasis or recurrence is still the main cause of death. Therefore, timely and early identification of EC, CRC and GC metastasis or recurrence can facilitate the timely diagnosis, treatment and prognosis of patients with these malignancies. CTCs have an important role in tumor metastasis and present significant prognostic value in several cancers.^[46–48]^ However, systematic analyses of EC, CRC and GC in gastrointestinal tumors have not been performed. This meta-analysis comprehensively summarizes relevant studies and provides strong evidence that the presence of CTCs in peripheral blood can predict disease progression and poor survival in patients with EC, CRC, and GC.

In this meta-analysis, the number of CTCs in peripheral blood was significantly associated with OS and PFS in EC, CRC, and GC patients. For OS, CTC-positive patients had a 2.05-fold higher risk of death than CTC-negative patients (95% CI: 1.75–2.40). For PFS, CTC-positive patients had a 2.27-fold higher risk of tumor progression than CTC-negative patients (95% CI: 1.79–2.89). Compared with the CTC-negative patients, the CTC-positive patients had a higher risk of death and tumor progression.

Although the prognostic value of CTCs in patients with various malignancies has been reported in many studies, a consensus has not been reached on the optimal sampling time for the collection of CTCs.^[49]^ Tina et al^[50]^ proposed that the presence of CTCs at baseline may serve as a surrogate marker of tumor burden; therefore, we took baseline as the sampling time. In this systematic review and meta-analysis, the results for an initial pool of 24 studies showed that the presence of CTCs has a strong predictive value for OS and PFS in EC, CRC, and GC and significantly increases the disease mortality and recurrence risks. However, a certain degree of heterogeneity was also observed during our meta-analysis. Therefore, we performed subgroup and meta-regression analyses to further evaluate groupings according to the study area, detection method, CTCs positivity cutoff, CTCs positivity detection rate, follow-up time, treatment modality, median patient age, tumor type, and literature score to identify the prognostic value of CTCs. The subgroup analysis showed that CTC-positive patients in all subgroups had a higher risk of death or tumor progression than CTC-negative patients. When grouped by study region, the risk of death and tumor progression for CTC-positive patients in the non-Asian region was similar to that in the Asian region, which may be related to the higher incidence of EC and GC in Asia and the higher incidence of CRC in Europe and the USA^[1]^ and the comparability of the studies included in this meta-analysis. When the results were stratified by detection method, the HRs for death and tumor progression of CTC-positive patients in the Asian and non-Asian groups were 2.05 and 2.67 and 2.02 and 3.31, respectively. Although the CellSearch system is the first standardized and FDA-approved semiautomated system for capturing and quantifying CTCs in peripheral blood,^[13]^ the results from the subgroup analyses suggested that methods other than CellSearch can improve the specificity of detection. In addition, clinical consensus remains equivocal regarding the optimal cutoff value for predicting the prognosis of CTCs in ESCC, CRC, and GC patients. Massimo et al^[51]^ noted that in CRC, CTCs values ≥3 are often used as the cutoff value. Moreover, a study on EC^[52]^ used CTCs values ≥5 as the optimal cutoff value. In our meta-analysis, most studies considered CTCs ≥3 as CTC-positive. After evaluating cutoff values of CTC ≥1, ≥2 and ≥3 in peripheral blood, we found that the cutoff value of CTCs ≥3 had a closer OS and PFS compared with cutoff values of ≥1 and ≥2 in peripheral blood (OS: HR 2.01 vs. 2.13 vs. 2.12, PFS: HR 1.57 vs. 3.07 vs. 3.49). Therefore, in this meta-analysis, we chose CTCs ≥3 as the cutoff value for CTC positivity. In addition, the increased risk of death and tumor progression for CTCs ≥3 and CTCs <3 were 1.85 and 2.42 and 1.59 and 3.21, respectively, suggesting that an increase in the number of CTCs could reduce the risk of OS and PFS. Furthermore, for OS, the meta-regression univariate analysis showed that the CTCs detection rate (*P* = .002) and tumor type (*P* = .007) were significant factors affecting disease OS while other covariates were not associated with heterogeneity. The multivariate analysis failed to identify the sources of heterogeneity in OS and PFS.

This meta-analysis is a systematic analysis of original studies that assessed peripheral blood CTCs in patients with gastrointestinal tumors (EC, CRC, and GC). Therefore, our results are more informative than those of previous studies. Our meta-analysis of 24 studies included 3803 EC, CRC, and GC cases, and it showed that compared with CTC-negative patients, CTC-positive patients had poorer OS and PFS, suggesting that the detection of CTCs has prognostic value for EC, CRC, and GC patients.

Certain limitations were observed in our meta-analysis. First, it is difficult to avoid or control for the influence of confounding factors, such as gender, age and race, in the meta-analysis due to the different original studies. For example, women are less susceptible to these malignancies while men are more susceptible. Moreover, the detection rate of EC and GC is higher in Asian countries while the detection rate of CRC is higher in European and American countries. Second, the meta-analysis used pooled data drawn from heterogeneous studies rather than raw data from individual patients, and we were unable to correct for all clinicopathological parameters according to consistent criteria. Third, some of the included literature did not explicitly provide HRs and 95% CI values, which should be extracted from relevant data and curves in the literature; otherwise, the prognostic effect should be assessed according to the method described by Tierney et al^[18]^ Fourth, our meta-analysis did not assess the prognostic value of CTCs in each clinical stage, i.e., TNM-stage patients. Fifth, this meta-analysis only included one study that using ISET, CMx platform, immune-magnetic or FACS-ICC to detect CTCs, respectively, which made the number of studies on detection methods other than CellSearch or RT-PCR very limited, so there are certain limitations. Finally, although our meta-analysis eliminated a portion of the heterogeneity of OS in the subgroup analysis, it did not identify the source of heterogeneity of PFS; moreover, the meta-regression multivariate analysis was not able to identify further sources of heterogeneity, which forced us to use a relatively conservative random effects model.

## 5. Conclusion

In conclusion, our meta-analysis shows that CTCs detection in peripheral blood may be an important tool for improving the prognosis of EC, CRC and GC patients, thus suggesting the possibility of conducting individualized treatment based on CTCs detection results in the future. However, prospective, well-designed, large-scale multicenter studies are needed to validate our results. Moreover, more high-quality RCTs must be performed to provide additional information, and using the same standardized detection platform and obtaining favorable CTCs numbers are expected to normalize and reduce inconsistencies between studies.

## Author contributions

Yuming Yao and Xiang Zhu wrote the main manuscript text and Jiayi Jiang prepared figures. Wei-xin Liu and Han Jiang mainly participated in research concept and design and data analysis. All authors reviewed the manuscript.

**Conceptualization:** Yuming Yao.

**Data curation:** Yuming Yao.

**Formal analysis:** Xiang Zhu, Yuming Yao.

**Methodology:** Xiang Zhu.

**Software:** Xiang Zhu.

**Supervision:** Jiayi Jiang, Weixin Liu.

**Visualization:** Han Jiang.
